# Analytical Characterization of an Inulin-Type Fructooligosaccharide from Root-Tubers of *Asphodelus* *ramosus* L

**DOI:** 10.3390/ph14030278

**Published:** 2021-03-19

**Authors:** Valentina Noemi Madia, Daniela De Vita, Antonella Messore, Chiara Toniolo, Valeria Tudino, Alessandro De Leo, Ivano Pindinello, Davide Ialongo, Francesco Saccoliti, Anna Maria D’Ursi, Manuela Grimaldi, Pietro Ceccobelli, Luigi Scipione, Roberto Di Santo, Roberta Costi

**Affiliations:** 1 Istituto Pasteur-Fondazione Cenci Bolognetti, Dipartimento di Chimica e Tecnologie del Farmaco, “Sapienza” Università di Roma, p.le Aldo Moro 5, 00185 Rome, Italy; valentinanoemi.madia@gmail.com (V.N.M.); valeria.tudino@uniroma1.it (V.T.); alessandro.deleo@uniroma1.it (A.D.L.); ivano.pindinello@uniroma1.it (I.P.); ialongo.1679357@studenti.uniroma1.it (D.I.); luigi.scipione@uniroma1.it (L.S.); roberto.disanto@uniroma1.it (R.D.S.); roberta.costi@uniroma1.it (R.C.); 2 Department of Environmental Biology, “Sapienza” University of Rome, p.le Aldo Moro 5, 00185 Rome, Italy; daniela.devita@uniroma1.it (D.D.V.); chiara.toniolo@uniroma1.it (C.T.); 3 D3 PharmaChemistry, Italian Institute of Technology, Via Morego 30, 16163 Genova, Italy; francesco.saccoliti@iit.it; 4 Department of Pharmacy, University of Salerno, Via Giovanni Paolo II, Fisciano, 84084 Salerno, Italy; dursi@unisa.it (A.M.D.); magrimaldi@unisa.it (M.G.); 5 Local Health Authority (ASL)/rm202, 00157 Rome, Italy; pietro.ceccobelli@libero.it

**Keywords:** *Asphodelus ramosus*, root-tubers, inulin, fructans, fructooligosaccharide, alkaline extraction, high-performance thin layer chromatography, NMR spectroscopy, 2D NMR analyses, infrared spectroscopy

## Abstract

Plant-based systems continue to play a pivotal role in healthcare, and their use has been extensively documented. *Asphodelus* L. is a genus comprising various herbaceous species, known by the trivial name Asphodelus. These plants have been known since antiquity for both food and therapeutic uses, especially for treating several diseases associated with inflammatory and infectious skin disorders. Phytochemical studies revealed the presence of different constituents, mainly anthraquinones, triterpenoids, phenolic acids, and flavonoids. Although extensive literature has been published on these constituents, a paucity of information has been reported regarding the carbohydrate composition, such as fructans and fructan-like derivatives. The extraction of water-soluble neutral polysaccharides is commonly performed using water extraction, at times assisted by microwaves and ultrasounds. Herein, we reported the investigation of the alkaline extraction of root-tubers of *Asphodelus ramosus* L., analyzing the water-soluble polysaccharides obtained by precipitation from the alkaline extract and its subsequent purification by chromatography. A polysaccharide was isolated by alkaline extraction; the HPTLC study to determine its composition showed fructose as the main monosaccharide. FT-IR analysis showed the presence of an inulin-type structure, and NMR analyses allowed us to conclude that *A. ramosus* roots contain polysaccharide with an inulin-type fructooligosaccharide with a degree of polymerization of 7–8.

## 1. Introduction

The genus *Asphodelus* L. belongs to the Liliaceae [1] or Asphodelaceae family according to APG IV classification of 2016 [2]. It is native to South Europe, Africa, the Middle East, and the Indian Subcontinent, which is where it is mainly distributed [3], and reaches its maximum diversity in the west of the Mediterranean, particularly in the Iberian Peninsula and in northwest Africa [4]. Plants of genus *Asphodelus* have been known since antiquity for their both therapeutic and food uses. Indeed, root-tubers are used as daily food, after being moistened and fried beforehand to eliminate the astringent compounds [5], and also the young stem, the leaves, and the roasted seeds [6]. Additionally, various ethnomedical uses were described for *Asphodelus* species, including the treatment of skin eczema or solar erythema, alopecia, paralysis, rheumatism, or earache [3]. *Asphodelus ramosus* L. is a species abundant in Mediterranean areas [5,7,8]. Its phytochemical studies revealed the presence of different constituents, mostly depending on the part of the plant. The roots mostly exhibit the presence of anthraquinones [9,10,11], while the aerial parts are mainly reported to have flavonoids and phenolic acids [12,13]. A paucity of information has been reported regarding carbohydrate composition [14,15], even though the members of *Asphodelus* genus are known to contain fructans [16], water-soluble carbohydrates consisting of repeating fructose. Here, we analyzed the water-soluble polysaccharide isolated from the alkaline extract of *A. ramosus*, resulting in an inulin-type fructan. The most common inulin-type fructans (ITFs) are inulin and fructooligosaccharides (FOSs), consisting of linear chains of fructose units linked by beta (2 → 1) fructosyl-fructose glycosidic bonds bound to a terminal glucose unit and a residue of glucose [17]. From a chemical point of view, FOS and inulin differ only in the degree of polymerization (DP): FOSs have a short chain with a DP between 2 and 9, while inulin can reach 60 units [18]. Inulin is widely used in industry because it improves the taste and mouthfeel of food and can replace fats in dairy and baked products [19]. Together with FOS, inulin is classified as a dietary fiber included in the class of nondigestible carbohydrates, since they cannot be hydrolyzed by intestinal enzyme; on the contrary, the microbiome can digest FOS and inulin giving several metabolites (i.e., short-chain fatty acids) [20] that gut microbiota and the host can utilize to improve gastrointestinal physiology with benefits on lipid metabolism, with decreased levels of serum cholesterol, triacylglycerols and phospholipids [21], and levels of glucose and insulin [22], immune function, and mineral absorption [23]. Other beneficial effects include host metabolism, and even in the development and homeostasis of the CNS [24]. Currently, FOS are increasingly included in food products and infant formulas due to their prebiotic effect, stimulating the growth of nonpathogenic intestinal microflora [21]. Inulin is used as a prebiotic, fat replacer, sugar replacer, texture modifier, and for the development of functional foods in order to improve health due to its beneficial role in gastric health. Indeed, inulin plays a preventive role against gastrointestinal complications such as constipation and many diseases of the intestinal tract, particularly irritable bowel diseases and colon cancer. Moreover, inulin consumption enhances the absorption of calcium, magnesium, and iron, and stimulates the immune system [25]. Interestingly, in vitro studies showed that inulin has radical scavenging activity and ferric reducing power, although they are weaker than vitamin C. Additionally, in vivo studies of laying hens showed that dietary supplementation with inulin significantly improved the antioxidant status of these animals [26].

Therefore, natural sources of inulin and FOS have great value, possessing health benefits for humans.

## 2. Results and Discussion

### 2.1. Extraction and Purification of Water-Soluble Polysaccharides from Root-Tubers of A. ramosus

Usually, the extraction of water-soluble polysaccharides is performed using hot water, at times assisted by microwaves [27] and ultrasound [28] to overcome some disadvantages, including the long time and low efficiency of extraction. The cooking processes were reported as a method to increase the efficiency of polysaccharide extraction from plant material, breaking hydrogen and hydrophobic bonds without the degradation of the covalent ones [29]. Therefore, we performed an extraction under high-pressure cooking treatment giving the solid called AR3. Nevertheless, since hot water extraction is associated with high extraction temperature, more time consumption, and low efficiency [30], an alternative way to increase extraction yield is alkaline extraction [31]. Therefore, we also performed an alkaline extraction of *A. ramosus* root-tubers, followed by the precipitation by ethanol of a brown solid (AR1).

A fast comparison by TLC of AR1 and AR3, using commercial inulin as a standard, allowed us to conclude that only AR1 contained water-soluble polysaccharide, as preliminarily confirmed by HPTLC analysis. Then, AR1 was purified by column chromatography on silica gel using an aqueous binary mobile phase (isopropanol:water), giving AR2.

### 2.2. High-Performance Thin-Layer Chromatography (HPTLC) Analysis

**Figure 1:**
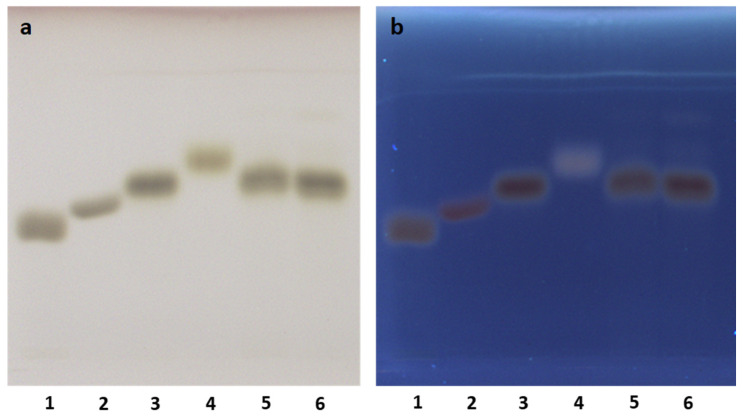
HPTLC analysis of the hydrolysate of the water-soluble polysaccharides from *A. ramosus* roots. Mobile phase: acetonitrile:water (85:15 *v/v*). (**a**) Visualization, WRT light, derivatization with a solution of sulfuric acid in methanol. (**b**) Visualization, UV 366 nm, derivatization with a solution of sulfuric acid in methanol. Tracks: 1. galactose (1 mg/mL; 8 μL); 2. glucose (1 mg/mL; 8 μL); 3 fructose (1 mg/mL; 8 μL); 4. arabinose (1 mg/mL; 8 μL); 5. hydrolyzed AR2 (1 mg/mL; 8 μL); 6. hydrolyzed inulin (1 mg/mL; 8 μL).

Commonly, the polysaccharides are subjected to hydrolysis followed by the determination of their monosaccharides by cleaving a glycosidic bond in a strong acid medium. Therefore, due to the instability of sugar monomers with strong mineral acids [32], milder hydrolysis is preferred. Here, we used an aqueous solution of trifluoroacetic acid (TFA) that seemed more advantageous than other ones reported in the literature, such as oxalic acid [33], because of the possibility to remove the residual TFA at the end of the hydrolysis process. Indeed, in our hands, the co-presence of residual oxalic acid influenced the retention factor (Rf) values of the monosaccharides (data not shown). The hydrolysis with TFA was previously investigated by Li et al. [29], giving the best conditions of temperature, time, and acid concentration to cleave the glycosidic bond and preserve the stability of the corresponding monomers. For the HPTLC analysis, in addition to fructose, the aldoses (glucose, galactose and arabinose) were used as references as, in a previous work [34], these monosaccharides were found to compose the mucilage isolated from tubers of *A. microcarpus* Salzm. and Viv. As the mobile phase, we chose a binary mixture (acetonitrile/water) where water is necessary to avoid diffused spots on the silica plate. Moreover, a sample of commercial inulin underwent hydrolysis with TFA in the same condition of AR2. After acidic hydrolysis of A. ramosus extract by TFA, the presence in the chromatogram of fructose is very evident from both AR1 (data not shown) and AR2 (Figure 1), evidenced as a brown spot visualized by derivatization with a solution of sulfuric acid in methanol. On the contrary, other monosaccharides were not found. This allowed us to conclude that AR2 is a fructose polymer.

### 2.3. Fourier-Transform Infrared Spectroscopy (FTIR) Analysis

The FT-IR spectrum (Figure S4) of AR2 shows a broad band due to the vibrations of hydroxyl groups (OH stretching) at 3275 cm^−1^ [35,36], while, in the region 1500-900 cm^−1^, the strongest bands at 1114 and 1020 cm^−1^ can be assigned to the stretching vibrations of C-OC- groups in furanosyl residues [35]. Lastly, the so-called “finger-print” region (1300-900 cm^−1^) can be useful for the characterization of a molecule since it is characteristic of every compound. The superposition of the inulin spectrum with that of AR2 (Figure S5) shows no significant differences in FT-IR spectra of commercial inulin and the saccharide extracted from A. ramosus.

### 2.4. Nuclear Magnetic Resonance (NMR) Studies

Data of ^1^H and ^13^C NMR, heteronuclear single quantum coherence (HSQC), and heteronuclear multiple bond correlation (HMBC) are compatible with inulin structure (Figure S2) and in accordance with the literature [35,37,38,39]. The ^1^H NMR spectrum (Figure S1) of AR2 showed the characteristic signals of polysaccharides in the region δ 3.40–5.40 ppm, with a higher magnitude below δ 4.20 ppm and a lower doublet in the anomeric region at 5.35 ppm (*J* = 3.8 Hz) related to H-1 proton of the α-Glc unit present. Intense signals were observed at more shielded fields: a doublet at 4.18 ppm (*J* = 8.8 Hz) and a triplet at 4.04 ppm (*J* = 8.8 Hz) related to H-3 and H-4 fructose, respectively, are present. The presence of fructosyl residues is also highlighted by ^13^C NMR and DEPT-135 spectra, whose signals are in agreement with literature data [35].

**Figure 2:**
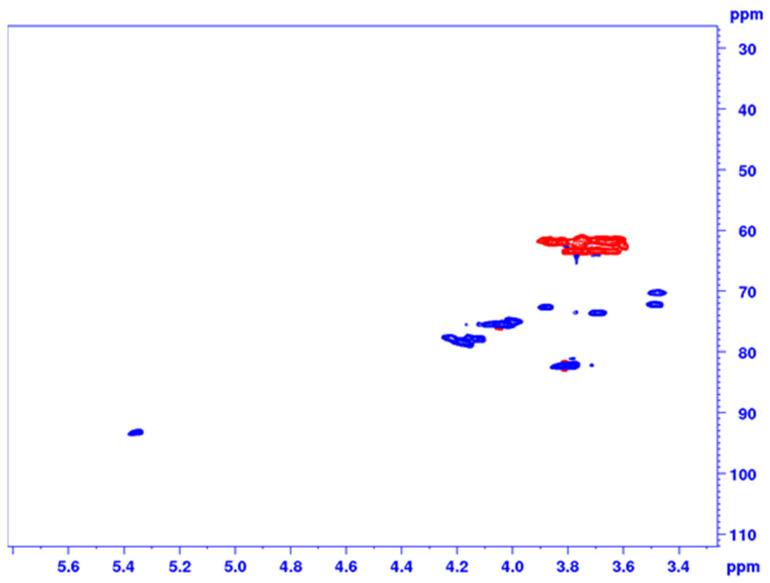
^1^H-^13^C multiplicity-edited HSQC spectrum of AR2.

^1^H-^13^C multiplicity-edited HSQC NMR spectrum of AR2 (Figure 2 and Figure S3) confirmed the assignments (Table 1). In details, the signals δ_H_ 4.18/δ_C_ 78.48, δ_H_ 4.04/δ_C_ 75.48, and δ_H_ 3.80/δ_C_ 82.20 can be assigned to C3, C4, and C5 of fructose, respectively, in agreement with literature data [35,39]. Moreover, the cross-peak δ_H_ 5.35/δ_C_ 93.38 belongs to the anomeric signal of glucose with an α-configuration, while the signal δ_H_ 3.75/δ_C_ 61.42 confirmed that glucose residue is in a terminal position of the chain.

**Figure 3:**
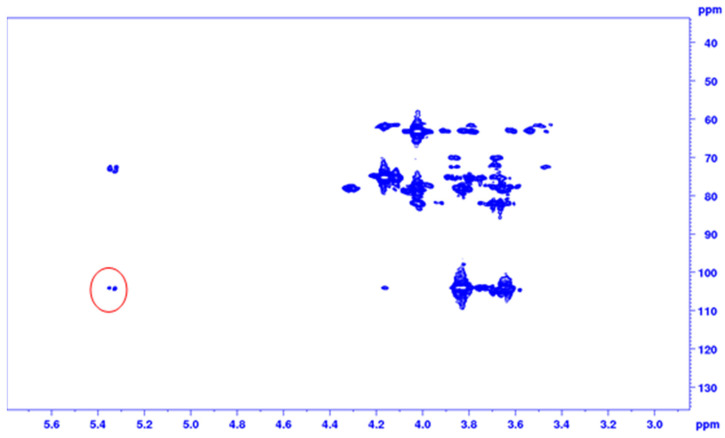
^1^H-^13^C 2D heteronuclear multiple bond correlation (HMBC) spectrum of AR2, correlation between the H-1 of the glucosyl residue and C-2 fructosyl residue is highlighted.

HMBC spectrum of AR2 showed the correlation between the H-1 of the glucosyl residue and C-2 fructosyl residue (Figure 3). Indeed, because of the cross-peak between H-1 of glucose and C-2 of fructose residue, glucose was confirmed to be terminally linked to a fructose chain with a non-reducing end [38].

Lastly, 2D NMR studies confirmed the (2 → 1) glycosidic bonds of fructosyl residue in the chain, in line with an inulin-type structure and in agreement with previous literature data [35,37,39].

To calculate the degree of polymerization (DP) of inulin, we followed the procedure previously described [40]. According to their experimental procedure, the degree of polymerization (DP) or the number of repeating units of a polymer can be calculated by comparing the ^1^H NMR signal intensity of a known moiety (typically, end-group(s) with a known number of protons) to that of the repeating chain unit of interest. By applying our data to the equation reported in the Material and Methods section, the DP of the polymer under scrutiny is found to be 7–8.

## 3. Materials and Methods

### 3.1. Materials

Roots of *A. ramosus* were collected in Vallecorsa (Lazio, Italy). Inulin, fructose, glucose, galactose, arabinose, trifluoroacetic acid, and deuterium oxide were purchased from Sigma-Aldrich (Milan, Italy). All chemical standards were of analytical grade. Concentrated sulfuric acid was obtained from Carlo Erba (Milan, Italy). Silica gel high-purity grade (pore size 60 Å, 220–440 mesh particle size, 35–75 μm particle size) were obtained from Sigma-Aldrich (Milan, Italy). Thin layer chromatography (TLC) was performed on Kieselgel GF254 plates and the plates were heated at 150–200 °C by spraying with KMnO_4_ solution until yellow coloration took place. The high-performance thin-layer chromatography (HPTLC) plates 10 × 10 cm with glass-backed layers silica gel 60 F_254_ (2 μm thickness) were purchased from Merck (Darmstadt, Germany) and prewashed by methanol. HPLC grade solvents were purchased from Sigma-Aldrich (Milan, Italy) and VWR (Milan, Italy). HPLC-grade water was prepared with a Milli-Q gradient (Millipore, Vimodrone, Italy) water purification system.

### 3.2. Extraction and Purification of the Water-Soluble Polysaccharides from Root-Tubers of A. ramosus

The alkaline solution was obtained as previously reported by us [41] by treating the fresh roots (1 kg) of *A. ramosus* with NaOH in pellets (100 g). Water-soluble polysaccharides were precipitated from the alkaline solutions by the addition of absolute ethanol as follows: 1 mL of alkaline solution was diluted with distilled water (1 mL). From the resulting solution, polysaccharides were precipitated by slow addition of absolute ethanol (ca. 5 mL). The solid was collected and dissolved with water; from the resulting solution, the solid was then precipitated with ethanol and dried under vacuum over P_2_O_5_ to constant weight. Fifty milligrams of a brown solid, called AR1, were obtained. Purification of AR1 was carried out on a gravity column using silica for flash chromatography as a stationary phase (weight ratio stationary phase: AR1 1:100). The mobile phase was a mixture of isopropanol:water 11:9 (*v/v*). The spot with the Rf equal to inulin was isolated and dried at low pressure to give a brownish solid, referred to as AR2, that was analyzed to elucidate its composition.

The aqueous extraction was performed with 10 g of dried root-tubers and hot water (1 L) under high-pressure cooking treatment, autoclaved for 10 min and left to rest for 24 h. An aliquot of the filtrate was concentrated under vacuum until the volume is reduced by 10 times and then added with 10-fold volume of ethanol. The precipitate was collected by centrifugation at 4000× g for 10 min, washed with ethanol 3 times, and dried until gaining a constant weight, obtaining the brownish solid AR3 (5% yield).

### 3.3. High-Performance Thin-Layer Chromatography Analysis

The samples (8 μL each) were applied with nitrogen flow by Linomat 5 sample applicator (CAMAG, Muttenz, Switzerland). The operating conditions were: syringe delivery speed, 10 s μL^−1^ (100 nL s^−1^); injection volume, 8 μL; band width, 6 mm; distance from bottom, 15 mm. The HPTLC plates were developed in the Automatic Developing Chamber 2 (ADC 2), the automatic and reproducibly developing chamber (CAMAG, Muttenz, Switzerland), saturated with the same mobile phase, acetonitrile:water (85:15 *v*/*v*), for 20 min at room temperature. The developed solvents (i.e., type of solvents and ratios) were carefully optimized before the analyses. The length of the chromatogram run was 70 mm from the point of application. The developed layers were allowed to dry on TLC Plate Heater III (CAMAG, Muttenz, Switzerland) for 5 min at 120 °C and then derivatized with sulfuric acid. Lastly, the plates were warmed for 5 min at 120 °C before inspection. All treated plates were then inspected under a UV light at 254 or 366 nm or under reflectance and transmission white light (WRT), respectively, at a Camag TLC visualizer (CAMAG, Muttenz, Switzerland), before and after derivatization. Aqueous solutions of AR1 and AR2 (4.5 mg/mL) were analyzed before and after hydrolysis with 3 mL of TFA (1 mg/mL) for 60 min at 90 °C, according to the procedure reported in the literature [29]. The hydrolyzed sample was evaporated to remove all the volatile components, taken up with water to obtain a final concentration of 1 mg/mL. Inulin, used as a reference, was subjected to the same acidic hydrolysis and reconstituted to the final concentration of 1 mg/mL. Aqueous solutions (1 mg/mL) of galactose, glucose, fructose, and arabinose were used as a reference.

### 3.4. FR-IR Analysis

FT-IR spectra of sample powders were collected using a FT-IR Perkin-Elmer Spectrometer One equipped with ATR (Attenuated Total Reflection) sampling device. Spectra were recorded over the spectral range of 400–4000 cm^−1^ at a 4 cm^−1^ resolution, coadding 32 scans. Before performing the analysis, the sample was dried under vacuum with P_2_O_5_ until gaining a constant weight.

### 3.5. NMR Experiments

For monodimensional experiments, 3 mg of AR2 were dissolved in 500 μL in 99.95% D_2_O. NMR spectra were recorded at 298 K and 400 MHz for ^1^H and 100 MHz for ^13^C on Bruker (Billerica, MA, USA) Avance 400 (Milano, Italy) spectrometer. Two-dimensional heteronuclear experiments were performed on 500 MHz Bruker DRX-500 spectrometer equipped with 5 mm BBI ^1^H-BB/^2^H Z-GRD probe, at 298 K. Two-dimensional ^1^H-^13^C heteronuclear multiple bond correlation (HMBC) and ^1^H-^13^C heteronuclear single quantum coherence (HSQC) spectra were acquired using hmbcgplpndqf (dummy scans 16, number of scans 92, time domain 256) and hsqcedetgpsp.3 (dummy scans 32, number of scans 80, time domain 256) pulse sequences available on Bruker software (Bruker, Wissembourg, France). ^1^H NMR and ^13^C resonances were assigned from the ^1^H-^13^C correlations observed in the. Chemical shifts were expressed in ppm and *J* values are shown in Hz. Quantitative measurements of signal intensity performed on both 1D and 2D experiments led to the DP calculation. In the 1D spectrum, we chose the isolated glucose unit signal at 5.35 ppm, and the fructose unit signal at 4.18 ppm. 

The DP was calculated using the following equation:

n_x_ = (a_x_ m_y_ n_y_) : (a_y_ m_x_) (1)

where a_x_ is the area or intensity of the ^1^H NMR peak of moiety x; n_x_ is the number of repeating units of moiety x; m_x_ is the number of protons of moiety x; a_y_ is the area or intensity of the ^1^H NMR peak of moiety y; n_y_ is the number of repeating units of moiety y; and m_y_ is the number of protons of moiety y. By applying our data to this equation, the DP of the polymer under scrutiny was found to be 7–8.

## 4. Conclusions

A polysaccharide was isolated from the root-tubers of *A. ramosus* by alkaline extraction and purified by column chromatography. HPTLC study was performed in order to determine the monosaccharide composition of the polysaccharide after acid hydrolysis. The results show fructose as the main monosaccharide into the polymer. The identity of the fructan was further confirmed by FT-IR analysis, where comparison of AR2 and inulin spectra showed the presence of the inulin-type structure. Furthermore, 1D and 2D NMR analyses allowed us to conclude that the water-soluble polysaccharide isolated from *A. ramosus* roots is an inulin-type fructan. Lastly, the degree of polymerization was calculated by ^1^H NMR, giving a DP of 7–8.

## Figures and Tables

**Table 1:** ^1^H and ^13^C NMR chemical shifts of AR2.

Residue	H-1/C-1 (ppm)	H-2/C-2 (ppm)	H-3/C-3 (ppm)	H-4/C-4 (ppm)	H-5/C-5 (ppm)	H-6/C-6 (ppm)
→1)-β-d-Fru*f*-(2→	3.85/3.66	-	4.18	4.04	3.80	3.78/3.70
	61.94	104.10	78.48	75.48	82.20	63.32
α-D-Glc*p*-(1→	5.35	3.49	3.69	3.48	3.88	3.75
	93.38	72.26	73.49	70.31	72.66	61.42

## Data Availability

Data are contained within the article.

## Supplementary Materials

The following are available online at https://www.mdpi.com/1424-8247/14/3/278/s1, Figure S1: ^1^H spectrum of AR sample in D_2_O, Figure S2: inulin structure, Figure S3: 2D ^1^H-^13^C-HSQC spectrum of AR sample in D_2_O, Figure S4: FT-IR spectrum of AR2 (neat), Figure S5: FT-IR spectra (neat) of AR2 (red) and standard inulin (black) in the region 1600–600 cm^−1^.
