# Supplementary material for: Analytical Characterization of an Inulin-Type Fructooligosaccharide from Root-Tubers of Asphodelus ramosus L
Source: Pharmaceuticals (Basel). 2021 Mar 19;14(3):278. doi: 10.3390/ph14030278 (PMC8003451; doi:10.3390/ph14030278)
Supplement: Supplementary file 1 [file pharmaceuticals-14-00278-s001.pdf]

Analytical Characterization of An Inulin-Type Fructooligosaccharide from Root-Tubers of *Asphodelus ramosus* L.

Valentina Noemi Madia [1,§], Daniela De Vita [2,§], Antonella Messore [1,*], Chiara Toniolo [2], Valeria Tudino [1], Alessandro De Leo [1], Ivano Pindinello [1], Davide Ialongo[1], Francesco Saccoliti [3], Anna Maria D'Ursi [4], Manuela Grimaldi [4], Pietro Ceccobelli [5], Luigi Scipione [1], Roberto Di Santo [1] and Roberta Costi[1]

[1] Istituto Pasteur-Fondazione Cenci Bolognetti, Dipartimento di Chimica e Tecnologie del Farmaco, "Sapienza" Università di Roma, p.le Aldo Moro 5, 00185 Rome, Italy; antonella.messore@uniroma1.it (A.M.); valentinanoemi.madia@gmail.com (V.N.M.); valeria.tudino@uniroma1.it (V.T.); alessandro.deleo@uniroma1.it (A.D.L.); ivano.pindinello@uniroma1.it (I.P.); ialongo.1679357@studenti.uniroma1.it; luigi.scipione@uniroma1.it (L.S.); roberto.disanto@uniroma1.it (R.D.S.); roberta.costi@uniroma1.it (R.C.)

[2] Department of Environmental Biology, "Sapienza" University of Rome, p.le Aldo Moro 5, 00185, Rome, Italy; daniela.devita@uniroma1.it (D.D.V.); chiara.toniolo@uniroma1.it (C.T.)

[3] D3 PharmaChemistry, Italian Institute of Technology, Via Morego 30, I-16163 Genova, Italy; francesco.saccoliti@iit.it

[4] Department of Pharmacy, University of Salerno, Via Giovanni Paolo II, Fisciano, 84084 Salerno, Italy; dursi@unisa.it (A.M.D.); magrimaldi@unisa.it (M.G.)

[5] ASL/rm202, Rome, Italy; pietro.ceccobelli@libero.it (P.C.)

* Correspondence: antonella.messore@uniroma1.it; Tel.: +39-06-4991-3965

§ These authors contributed equally

Contents

Figure S1: ^{1}H spectrum of AR2 sample in D_2O;

Figure S2: inulin structure;

Figure S3: 2D ^{1}H-^{13}C-HSQC spectrum of AR2 sample in D_2O.

Figure S4: FT-IR spectrum of AR2 (neat);

Figure S5: FT-IR spectra (neat) of AR2 (red) and standard inulin (black) in the region 1600-600 cm^{-1}.

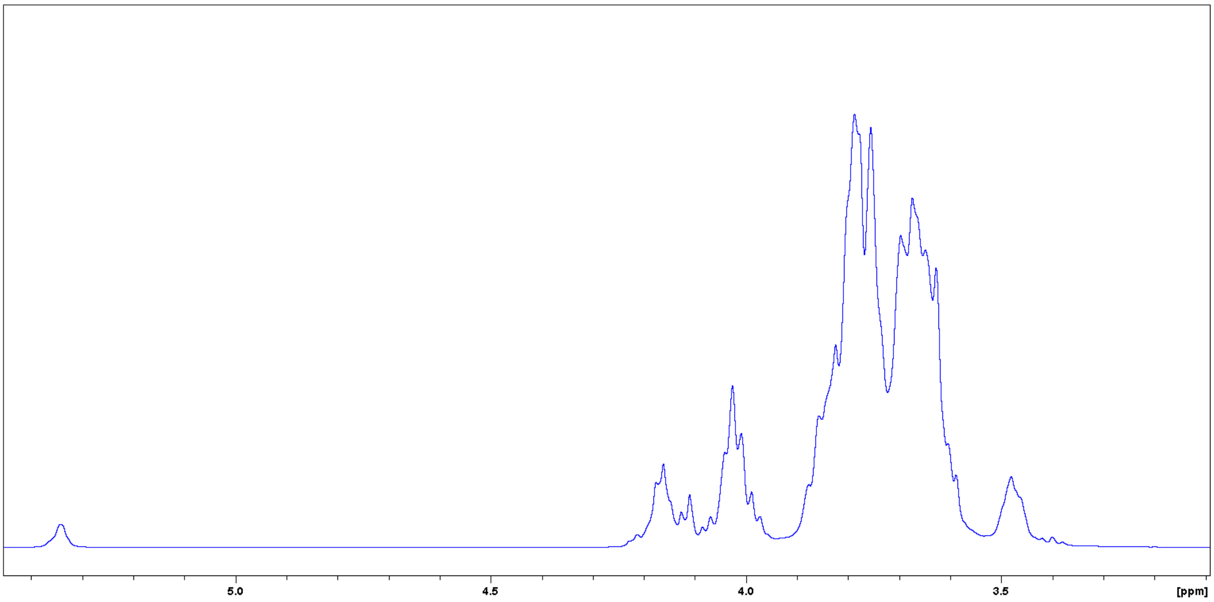

Figure S1. ^{1}H spectrum of AR2 sample in D_2O (500 μL) (Bruker 500 MHz, Temp. 298 K).

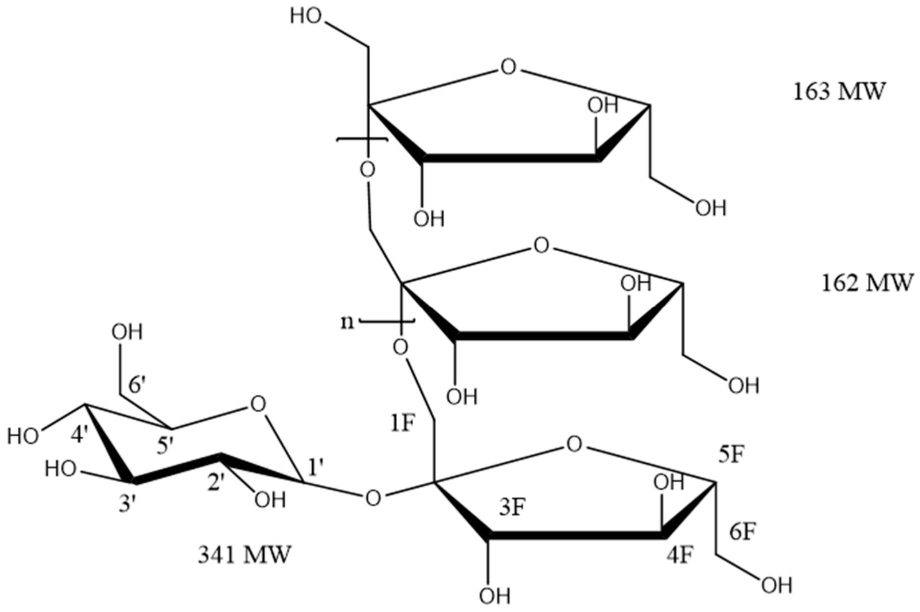

Figure S2. Inulin structure

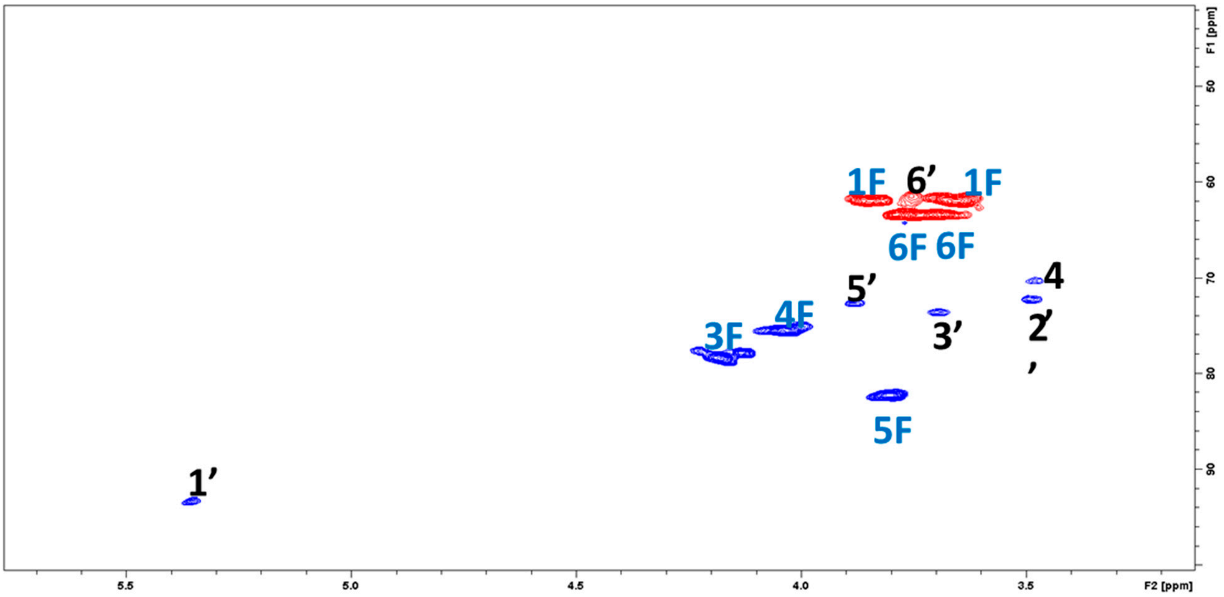

Figure S3. 2D ^{1}H-^{13}C-HSQC spectrum of AR2 sample in D_2O (500µL) (Bruker 600 MHz, Temp. 298 K).

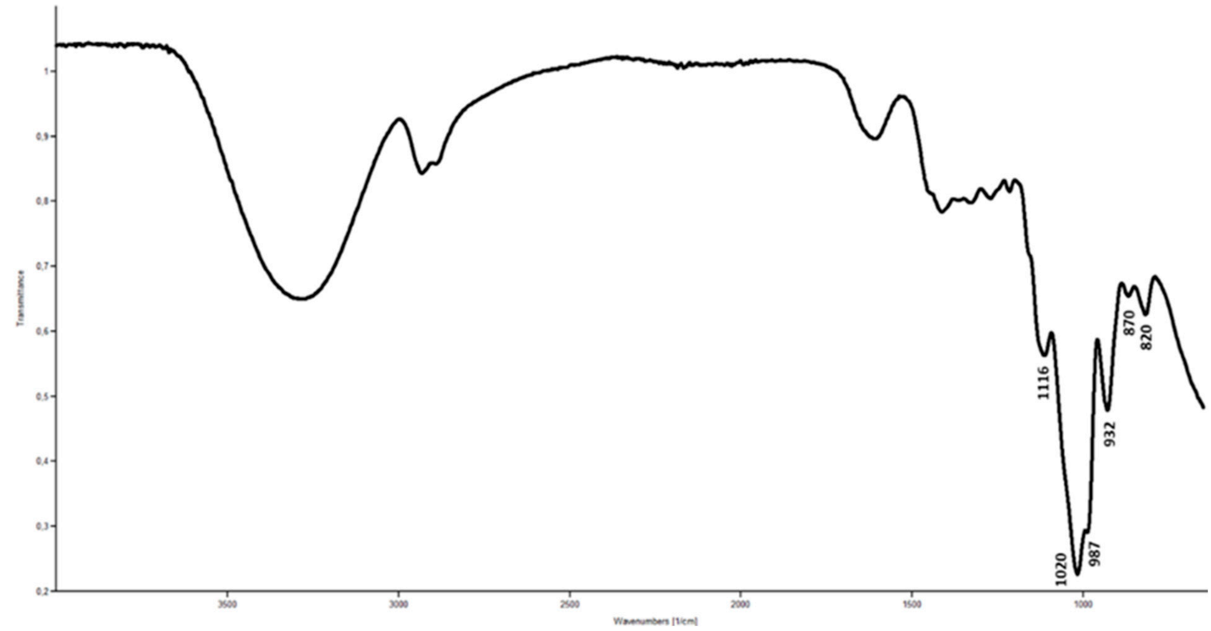

Figure S4. FT-IR spectrum of AR2 (neat)

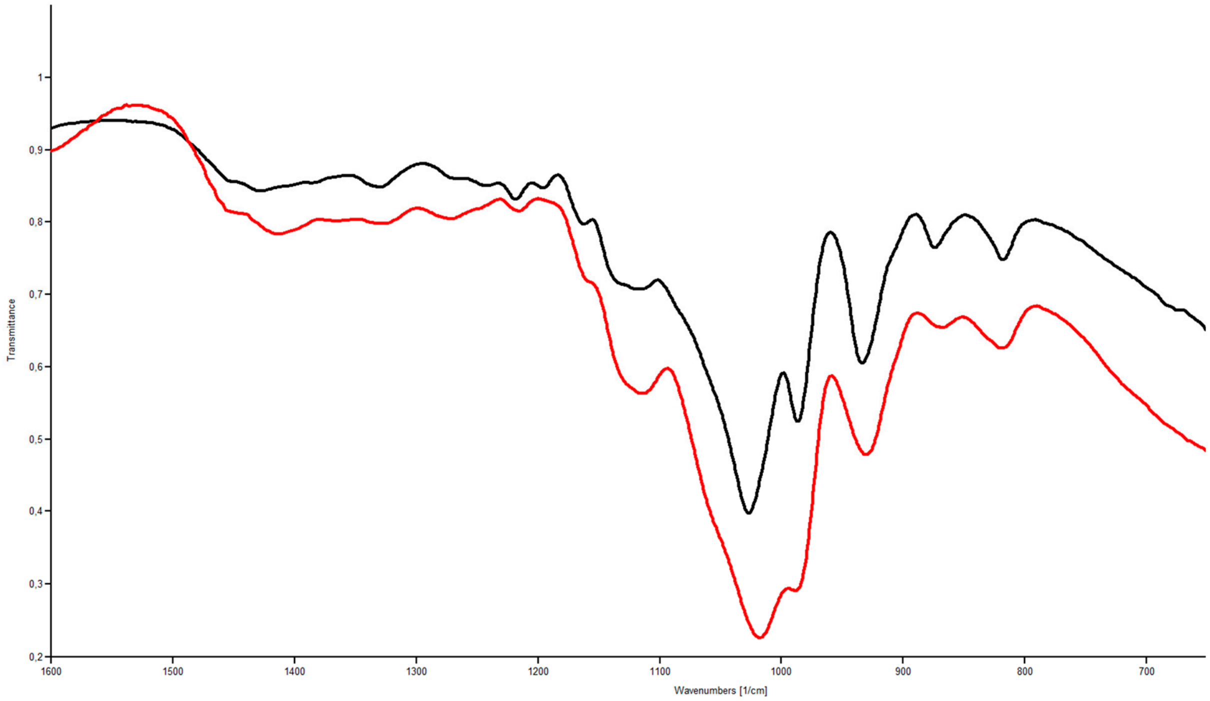

Figure S5. FT-IR spectra (neat) of AR2 (red) and standard inulin (black) in the region 1600–600 cm^{-1}.
